# Sexual Functioning and Patient-Reported Concerns After Stroke: An Integrated Mixed-Methods Study in Clinical Rehabilitation

**DOI:** 10.3390/nursrep16070243

**Published:** 2026-07-14

**Authors:** Alfredo Manuli, Maria Grazia Maggio, Andrea Calderone, Lilla Bonanno, Provvidenza Tomasello, Caterina Pucci, Morena De Francesco, Gianluca Pucciarelli, Rocco Salvatore Calabrò

**Affiliations:** 1 Department of Biomedicine and Prevention, University of Rome Tor Vergata, 00133 Rome, Italy; manulialfredo@gmail.com (A.M.); g.pucciarelli81@gmail.com (G.P.); 2 IRCCS Centro Neurolesi Bonino-Pulejo, S.S. 113 Via Palermo, C. da Casazza, 98124 Messina, Italy; andrea.calderone@irccsme.it (A.C.); lilla.bonanno@irccsme.it (L.B.); enza.tomasello@irccsme.it (P.T.); roccos.calabro@irccsme.it (R.S.C.); 3 S. Anna Institute, Via Siris 11, 88900 Crotone, Italy; puccicaterina88@gmail.com; 4 Institute of Bioimaging and Complex Biological Systems (IBSBC), National Research Council of Italy (CNR), 88100 Catanzaro, Italy; morenadefrancesco00@cnr.it

**Keywords:** stroke, sexual function, sexual health, rehabilitation, mixed-methods, nursing, quality of life, depression, relationship functioning

## Abstract

**Background/Objectives:** Sexual health is frequently under-addressed after stroke, despite its relevance to intimacy, identity, relationships, and rehabilitation. This convergent mixed-methods study integrated sex-specific screening with descriptive narrative material to examine sexual functioning and patient-reported concerns in clinical rehabilitation. **Methods:** Twenty-six adults were assessed: ten with the Female Sexual Function Index (FSFI), sixteen with an International Index of Erectile Function six-item field (IIEF-6), and ten with analyzable semi-structured interview responses. The sex-specific measures were analyzed separately. Continuous variables were summarized by median and interquartile range; nonparametric correlations and group comparisons underwent Benjamini–Hochberg false discovery rate correction. Quantitative and qualitative findings were integrated in a participant-level joint display. This integration yielded case-level meta-inferences rather than inferential mixed-methods testing in this sample. **Results:** Nine of ten FSFI profiles and nine of sixteen IIEF-6 profiles met the respective exploratory threshold for possible dysfunction. No association or group comparison remained statistically significant after correction. Six of eighteen threshold-positive profiles had analyzable interviews, twelve represented narrative silence, and four interviewed participants were threshold-negative. Narratives documented fear, embarrassment, reduced desire or sexual frequency, relational or communication change, support needs, clinical sequelae, and positive or neutral adaptation. Screening and narratives showed convergence, complementarity, and divergence: low scores did not establish personal distress, whereas concerns could occur above a threshold. **Conclusions:** Responsive sexual rehabilitation should combine confidential, permission-based, patient-led screening and narrative inquiry with individualized information, optional support, appropriate referral, and reassessment according to readiness. These preliminary, hypothesis-generating findings do not validate an intervention or support confirmatory inference.

## 1. Introduction

Stroke remains one of the leading causes of long-term adult disability worldwide, and its burden extends far beyond motor impairment, communication loss, and dependence in activities of daily living [1]. Sexual functioning is one of the domains most frequently altered after stroke, yet it is still treated as a marginal concern within many rehabilitation pathways. Post-stroke sexual dysfunction may involve reduced desire, impaired arousal, erectile dysfunction, vaginal lubrication difficulties, orgasmic changes, pain, reduced satisfaction, and avoidance of sexual activity [2,3]. These manifestations are clinically heterogeneous because sexuality is shaped by neurological injury, psychological adjustment, relational context, body image, medication exposure, and cultural meanings. For this reason, sexual health after stroke should not be reduced to genital function alone. It represents a multidimensional aspect of recovery that intersects with identity, intimacy, autonomy, quality of life, and participation.

Evidence focusing on women after stroke shows that sexual concerns may affect desire, arousal, lubrication, orgasm, satisfaction, and pain, while the available rehabilitation literature remains limited and uneven [4]. Cohort data from rehabilitation settings also suggest that sexual disorders are common and that many patients report unmet needs for information or professional discussion during recovery [5]. In men, erectile dysfunction is particularly visible; however, recent work indicates that age, vascular burden, and pre-existing cardiovascular risk may interact with post-stroke changes in complex ways [6]. These sex-specific manifestations support the use of instruments that respect biological and experiential differences rather than forcing all patients into a single generic score.

The clinical relevance of sexual functioning is amplified by its association with quality of life and psychosocial adaptation. Recent cross-sectional evidence indicates that many individuals after stroke experience sexual dysfunction together with disability, pain, positioning difficulties, role changes, self-esteem concerns, and depressive symptoms [7]. A biopsychosocial study of patients admitted to specialized cognitive rehabilitation similarly found that sexual satisfaction was associated with emotional and relational factors, not only with neurological severity [8]. These findings are consistent with a broader rehabilitation perspective in which sexual recovery is influenced by mood, fatigue, fear of recurrence, altered body image, communication difficulties, and the perceived reactions of partners. Sexuality may therefore become a sensitive indicator of how the person integrates the stroke into daily life, couple functioning, and self-perception. When this domain is ignored, patients may interpret silence as a sign that sexual concerns are irrelevant, unsafe to disclose, or outside the remit of rehabilitation. This silence can increase shame and leave couples to negotiate uncertainty without clinical guidance. A comprehensive assessment should therefore recognize sexual health as part of functional recovery rather than as an optional adjunct to it. Nurses are therefore well placed to identify concerns during routine rehabilitation encounters and follow-up.

Despite this relevance, sexuality is rarely assessed systematically in stroke care. A study in a Latin American reference hospital found that sexual dysfunction affected more than half of stroke survivors and that physicians reported multiple barriers to addressing the topic [9]. Reviews and clinical perspectives have emphasized that patients often wait for professionals to initiate the conversation, while professionals may avoid it because of limited training, discomfort, lack of time, uncertainty about roles, or fear of causing embarrassment [10]. Similar barriers have been reported among physiotherapists working with stroke survivors, where attitudes and practice patterns show a persistent gap between recognition of need and routine clinical action [11]. These barriers are not merely organizational. They also reflect cultural taboos around sexuality, assumptions that older or disabled people are not sexually active, and a tendency to prioritize visible impairments over intimate outcomes.

Rehabilitation professionals, including nurses, themselves acknowledge this gap. International survey data show that knowledge, comfort, and timing of training influence whether clinicians provide sexuality-related rehabilitation after stroke [12]. Systematic review evidence further suggests that interventions used by allied health professionals remain limited, although structured education, counselling approaches, pelvic floor training, and interview guides may help clinicians identify concerns and provide appropriate support [13]. A Cochrane review concluded that the evidence base for interventions remains small and uncertain, which reinforces the need for studies that characterize both measurable dysfunction and the lived meaning of sexual change [14].

Patient perspectives provide an essential corrective to purely biomedical accounts. Qualitative and patient-centered studies show that stroke survivors want clear information, permission to discuss sexual activity, reassurance about safety, and acknowledgement of changes in confidence, attraction, intimacy, and partnership [15]. A qualitative synthesis of stroke survivors and partners highlighted that sexuality after stroke is often experienced as a disruption of identity and relational connection, not simply as a performance problem [16]. Multi-site qualitative work on sexual rehabilitation similarly points to the need for services that are individualized, interdisciplinary, and sensitive to the timing of recovery [17]. These findings justify approaches that can connect standardized measurement with narrative data.

Mixed-methods research is particularly suited to this field because sexual functioning after stroke includes domains that can be quantified and meanings that require interpretation. Interview-based tools have already shown feasibility for opening conversations about sexuality in stroke rehabilitation [18]. Trial evidence also suggests that structured sexual rehabilitation programmes can be delivered, although further research is needed to clarify for whom, when, and how these interventions should be integrated into routine care [19]. Earlier literature reviews called for a more comprehensive assessment of sexuality after stroke, including medical, psychological, relational, and rehabilitation dimensions [20].

The present study addresses this gap by examining sexual functioning after stroke in a clinical rehabilitation setting using a convergent mixed-methods design. The quantitative component evaluates sex-specific sexual function together with depressive symptoms, dyadic adjustment, family relationships, health-related quality of life, functional status, and clinical variables. The qualitative component explores patients’ perceptions of post-stroke changes in sexuality, identity, body image, intimacy, partner communication, barriers, and rehabilitation needs. The study aims to identify the domains most affected, examine associations between sexual functioning and psychosocial or clinical variables, and integrate numeric and narrative evidence. Here, responsive sexual rehabilitation denotes a confidential, permission-based, patient-led, and iterative clinical process that combines sex-specific screening with narrative inquiry; explores physical, emotional, relational, and contextual meanings; and aligns individualized information or nursing and interdisciplinary support with the patient’s preferences, readiness, and rehabilitation stage. The term describes an empirically informed clinical process rather than a validated intervention.

## 2. Materials and Methods

### 2.1. Study Design

This study used a convergent parallel mixed-methods design within a clinical rehabilitation assessment pathway [21,22]. Quantitative and qualitative materials were collected during the same clinical pathway, analyzed separately, and integrated at the interpretation stage through side-by-side comparison and joint-display logic [23,24]. The design was retained because post-stroke sexuality includes measurable domains, such as sexual performance, mood, relationship functioning, and quality of life, as well as subjective experiences related to identity, intimacy, body image, fear, and communication.

**Figure 1:**
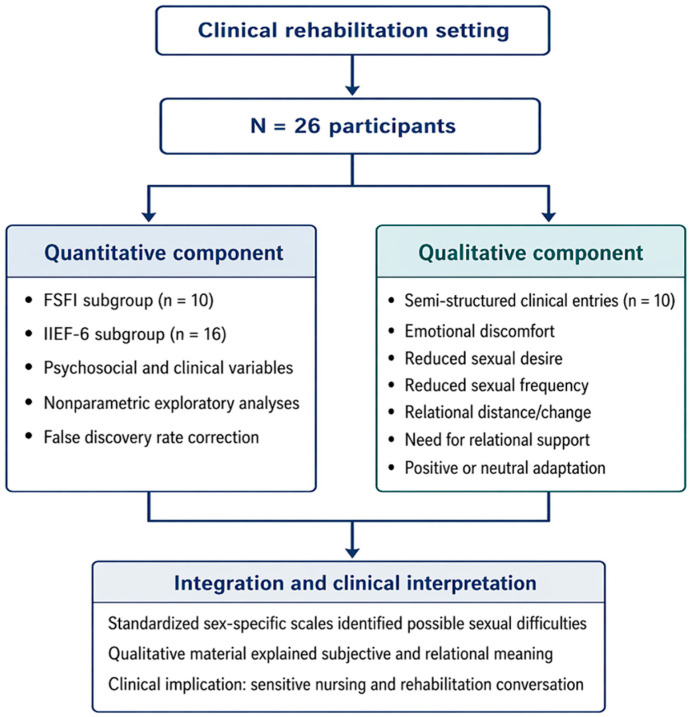
Mixed-methods study design and integration workflow. The figure summarizes the convergent mixed-methods structure of the study. Participants were assessed in a clinical rehabilitation setting and contributed to sex-specific quantitative assessment, qualitative semi-structured interview responses, or both, according to data availability. The quantitative strand included FSFI data for participants with female sexual-function data, International Index of Erectile Function (IIEF-6) data for participants with male erectile-function data, psychosocial and clinical variables, and nonparametric analyses with false discovery rate correction. The qualitative strand included semi-structured clinical entries describing emotional, sexual, relational, and adaptive experiences. Integration was performed through descriptive joint-display synthesis to compare convergence, complementarity, divergence, and silence between the two strands. Abbreviations: FSFI, Female Sexual Function Index; IIEF, International Index of Erectile Function.

The qualitative strand remained part of the mixed-methods design, but it was framed more cautiously as a descriptive, clinical thematic component. It was based on a semi-structured interview guide and structured case report forms rather than audio-recorded verbatim transcripts. Accordingly, the analysis did not claim qualitative saturation or deep phenomenological interpretation; instead, it was used to contextualize the quantitative findings and to identify clinically relevant meanings that could not be inferred from standardized scores alone. The mixed-methods study design and integration workflow are summarized in Figure 1.

### 2.2. Setting and Participants

Participants were adults assessed in a hospital-based rehabilitation clinical setting under a post-stroke or stroke-related cerebrovascular rehabilitation pathway. The database included 26 participants with demographic, clinical, sexual function, mood, relational, family, quality-of-life, and clinical descriptor data. Age, neuroimaging or clinical event description, etiology coding, date of event, date of enrollment, civil status, relationship duration, parental status, education, years of schooling, and medication exposure were recorded. The time from event to enrollment was calculated when both dates were available and was retained as a continuous descriptor because the sample included participants at different rehabilitation stages after the index event. Eligibility required adult age, capacity to provide informed consent, sufficient communicative ability to complete the questionnaires or semi-structured interview fields, and a documented acquired cerebrovascular or stroke-related event recorded in the clinical rehabilitation records. Ischemic, hemorrhagic/hematoma, and clinically coded stroke-related records were retained as part of the same rehabilitation pathway; event-type heterogeneity was treated as a descriptive limitation rather than as a basis for subgroup inference. Participants were excluded when the assessment could not be completed because of insufficient consent capacity or inability to provide usable clinical and questionnaire data.

### 2.3. Sampling and Recruitment

A consecutive sampling strategy was used. Eligible patients attending the clinical rehabilitation pathway were invited to participate during routine assessment. Recruitment did not generate a separate partner-level analytic sample in the available database. Partner or caregiver impressions were documented only as contextual interview fields when available, and these data were treated as part of the qualitative contextual material rather than as independent dyadic observations.

### 2.4. Quantitative Measures

Sexual functioning was assessed using sex-specific instruments. Female sexual function was recorded through the Female Sexual Function Index (FSFI), with domain raw sums for desire, arousal, lubrication, orgasm, satisfaction, and pain available in the database [25]. Because the database stored domain-level values, FSFI domain scores were calculated using the standard FSFI domain weighting factors before deriving the total score [25]. Item-level FSFI responses were not available and therefore could not be independently audited. A total FSFI score below 26.55 was used descriptively to classify possible female sexual dysfunction. Male sexual functioning was recorded through an IIEF-6 derived brief measure labelled IIEF-6 in the database, with a total score available when applicable [26]. An IIEF-6 score of 16 or lower was used as a conservative descriptive threshold for possible moderate-to-severe erectile dysfunction. Because FSFI and IIEF-6 scores are not metrically interchangeable, sexual function was planned for description and analysis using parallel sex-specific outcomes rather than a single pooled sexual function score.

Depressive symptoms were assessed using the Hamilton Depression Rating Scale (HAM-D) total score [27]. Health-related quality of life was assessed using the 12-item Short Form Health Survey, with physical, mental, and total scores recorded in the database [28]. Relationship functioning was assessed with the Dyadic Adjustment Scale fields available in the database, including consensus on important issues, satisfaction with the relationship, shared activities, satisfaction with affective and sexual life, and total score [29]. Family relationship functioning was assessed with the Family Relationship Index, including cohesion, communication, conflict, and total score [30]. A field labelled Ranking Scale was present in the database; however, because the available values were not supported by a definitive coding legend and were not informative for analysis, this field was not used as an analytic variable. Clinical variables included event date, enrollment date, neuroimaging or event description, etiology code, medication exposure, and education-related variables.

### 2.5. Qualitative Component

The qualitative component was based on the semi-structured interview developed within a clinical research tradition on sexual functioning in neurological populations, including epilepsy, multiple sclerosis, and severe spasticity management [31,32,33]. The interview was designed to explore multiple dimensions of sexuality after stroke, including changes in sexual functioning, intimacy, body image, emotional experiences, partner relationships, communication, perceived attractiveness, barriers to sexual activity, and rehabilitation needs.

Interviews were conducted by trained clinicians following the same interview guide to ensure consistency across participants. Participants’ responses were systematically documented in structured case report forms during the interview and subsequently entered into the study database. Meaningful interview material was available for a subset of 10 participants. Although interviews were not audio-recorded and verbatim transcripts were unavailable, the qualitative dataset consisted of systematically documented semi-structured interview responses rather than routine clinical notes.

Qualitative data were analyzed using a pragmatic thematic approach [34,35]. The documented responses were reviewed repeatedly to achieve familiarization with the material and to identify meaningful units related to sexuality, emotional experiences, relationship adaptation, support needs, clinical consequences, and adaptive coping. These units were iteratively grouped into candidate themes, organized into thematic matrices, checked against the source material, and reviewed by the research team until descriptive consensus was reached. Because verbatim transcripts were unavailable, the findings are presented as a descriptive thematic analysis rather than a phenomenological analysis, and no claim of theoretical or data saturation was made. The instruments, data sources, and their analytical roles are summarized in Table 1.

### 2.6. Procedure and Data Collection

After eligibility verification and consent, participants completed the quantitative assessment and the semi-structured interview fields during the clinical evaluation pathway. The quantitative measures were administered according to the sex-specific applicability of each sexual function scale and according to the availability of relational, family, mood, quality of life, and clinical fields. Clinical and demographic information was extracted from the study database. Qualitative responses were entered into open-text fields linked to the participant record. Confidentiality was maintained by using participant identifiers rather than names in the analytic dataset.

### 2.7. Outcomes and Variables

The primary quantitative outcome was post-stroke sexual functioning assessed through sex-specific measures, namely FSFI scores for participants with female sexual function data and IIEF-6 total score for participants with male erectile function data. Secondary outcomes were depressive symptoms, relationship adjustment, family relationship functioning, health-related quality of life, time from event to enrollment and additional exploratory clinical descriptors. Qualitative domains, derived from the semi-structured interview guide, were changes in sexuality, desire, arousal, sexual activity, attractiveness, identity, emotional state, partner communication, relational adaptation, barriers to intimacy, and perceived rehabilitation needs.

### 2.8. Statistical Analysis and Mixed-Methods Integration

Quantitative analyses were conducted on the available case sample of 26 participants. Because sexual functioning was assessed using sex-specific instruments, FSFI and IIEF-6 outcomes were analyzed separately and were not combined into a single composite score. FSFI domain raw sums were checked for range plausibility and converted to standard weighted domain scores before analysis. The FSFI total score was calculated as the sum of weighted domain scores so that the resulting total score was consistent with the expected FSFI scoring range. Because item-level FSFI responses were unavailable, the reconstructed score was treated as a domain-level estimate and interpreted cautiously.

The distribution of continuous variables was assessed using the Shapiro–Wilk test. Given the small sample size, skewed distributions, and exploratory nature of the quantitative strand, nonparametric methods were used for inferential analyses. Continuous variables were summarized as median and interquartile range (IQR), and categorical variables were described as frequencies and percentages. Associations between sex-specific sexual-function scores and demographic, relational, family, mood, quality-of-life, and exploratory clinical variables, including time from event to enrollment, were examined using Spearman rank correlation coefficients. To account for multiple testing, *p*-values were adjusted using the Benjamini–Hochberg false discovery rate (FDR) procedure. FDR-adjusted *p*-values are reported as pFDR, defined as the *p*-value after Benjamini–Hochberg correction. Exploratory group comparisons of FSFI total and IIEF-6 scores were performed using the Wilcoxon rank-sum test for two-group comparisons and the Kruskal–Wallis test for comparisons involving more than two groups. These tests were retained for transparency but not interpreted as confirmatory because of the very small subgroup sizes. Additional exploratory analyses were conducted on FSFI domain scores using the same nonparametric approach. Variables derived from the narrative neuroimaging field were recoded into simplified categories, including lesion side and broad event type, and were treated as exploratory clinical descriptors.

Qualitative data were analyzed using a pragmatic thematic approach. Open-text and clinically relevant short-answer responses were manually reviewed to identify recurrent themes related to sexual change, emotional experience, intimacy, relational adaptation, perceived barriers, and support needs. Quantitative and qualitative findings were integrated using a participant-level joint display to compare convergence, complementarity, divergence, and narrative silence between standardized scores and documented interview responses [23,24]. The display included every participant meeting an exploratory sex-specific threshold and every participant with analyzable interview material, with one pseudonymized row per unique participant. FSFI and IIEF-6 scores remained separate, and within-instrument ordering was descriptive only. Given the small qualitative subset, integration supported cautious case-level meta-inferences rather than inferential mixed-methods testing. All analyses were performed using R version 4.4.2. Reporting was planned with attention to observational, qualitative, and mixed-methods guidance, including Strengthening the Reporting of Observational Studies in Epidemiology and Good Reporting of A Mixed Methods Study principles [36,37].

### 2.9. Ethical Considerations and Declarations

The study was conducted in accordance with the principles of the Declaration of Helsinki. The study protocol, registered at ClinicalTrials.gov (NCT06278363; Sexual Nursing Care in Stroke Patients, StrokeSex_23), was approved by the Local Ethics Committee of IRCCS Bonino-Pulejo (Approval Number: 06/2023). Participants were considered eligible only if they were able to provide informed consent and complete the clinical assessment procedures. Data were de-identified for analysis, and qualitative material was handled as sensitive health information because sexual functioning, relationship adaptation, and partner perceptions are private domains.

## 3. Results

### 3.1. Sample Characteristics

The quantitative sample comprised 26 participants. Descriptive characteristics are reported in Table 2 as median [IQR], and additional categorical characteristics are reported in Supplementary Table S1. The sample included 15 participants with etiological code 2 (57.69%) and 11 with etiological code 1 (42.31%). Pharmacological treatment data were available for 22 participants: 15 reported ongoing pharmacological treatment (68.18%), whereas 7 reported no ongoing treatment (31.82%). Lesion side, derived from the narrative neuroimaging field, was classified as right-sided in 13 participants (50.00%), left-sided in 5 (19.23%) and unclear in 8 (30.77%). The broad event type was retained as a descriptive clinical descriptor and was not used to support subgroup inference. Sex-specific sexual-function measures were available for 10 participants for FSFI and 16 participants for IIEF-6.

### 3.2. Correlates of Female Sexual Function

In the FSFI subgroup (*n* = 10), none of the Spearman correlation analyses remained statistically significant after FDR correction. For transparency, the numerically largest uncorrected coefficient was observed between the FSFI total score and satisfaction with affective and sexual life within the dyadic relationship (rho = 0.54, *p* = 0.105, pFDR = 0.82). However, this result was not interpreted as evidence of an association because the adjusted probability value was clearly above the threshold for statistical significance. The remaining correlations with demographic, relational, family, mood, quality-of-life, and time-from-event variables were also non-significant after FDR correction. Therefore, the FSFI analyses were considered descriptive and hypothesis-generating only.

### 3.3. Correlates of Male Erectile Function

In the IIEF-6 subgroup (*n* = 16), no Spearman correlation remained statistically significant after correction for multiple testing. The numerically largest uncorrected coefficient involved age (*rho* = −0.45, *p* = 0.082, *p*FDR = 0.70). This value was reported only to ensure transparent presentation of the exploratory analyses and was not interpreted as evidence of an age-related association with erectile-function scores. Correlations involving relationship functioning, family variables, depressive symptoms, health-related quality of life, and time from event to enrollment were similarly non-significant after FDR correction. These findings indicate that the present sample did not provide statistically supported evidence of quantitative correlates of IIEF-6 scores.

### 3.4. Exploratory Group Comparisons

Exploratory nonparametric group comparisons of sex-specific total sexual-function scores did not show any statistically significant difference after FDR correction. Group-wise descriptive summaries are provided in Supplementary Table S2, and exploratory comparisons are reported in Supplementary Table S3. Because several cells contained very small numbers of participants, these comparisons were retained only as transparent descriptive checks and were not interpreted as evidence for lesion-side, event-type, or clinical subgroup effects.

### 3.5. Qualitative Results

Meaningful semi-structured interview material was available for 10 participants. The material consisted of systematically documented responses recorded in structured case report forms rather than routine clinical notes or full verbatim transcripts. Accordingly, findings were interpreted descriptively at the participant level, with no claim of saturation. The narratives showed heterogeneous post-stroke sexual and relational experiences (Table 3). Fear, performance anxiety, or embarrassment was documented in three participants; reduced desire-related material in three; and an explicit decrease in sexual frequency in one. Individual records also documented relational distance or communication change, relational accommodation needs, and clinical or physical context. Several participants described serenity, tranquility, well-being, or no specific sexual problem, underscoring that low sexual-function scores did not uniformly imply subjective distress. Themes were non-mutually exclusive and were not interpreted as prevalence estimates. A simplified map is provided in Supplementary Figure S1.

### 3.6. Integration of Quantitative and Qualitative Findings

Using the predefined exploratory thresholds applied to the corrected sex-specific measures, 9 of 10 FSFI profiles (90.0%) and 9 of 16 IIEF-6 profiles (56.3%) were threshold-positive. These proportions were not pooled because the instruments are sex-specific and not metrically interchangeable, and threshold status was not interpreted as a diagnosis or as evidence that difficulty began after stroke. Participant-level linkage with the 10 analyzable interviews showed that 6 of the 18 threshold-positive profiles had narrative material (five FSFI profiles and one IIEF-6 profile), whereas 12 of 18 represented narrative silence. Four of the 10 interviewed participants were threshold negative. The union of threshold-positive and interviewed cases therefore comprised 22 unique participants in the joint display (Table 4).

Integration was descriptive and case-linked rather than confirmatory. Convergence was present when the threshold status and the documented narrative pointed in the same direction. Complementarity occurred when narratives added fear, embarrassment, personal meaning, communication change, clinical context, or support needs that a score could not explain. Divergence was visible when low sexual-function scores coexisted with serenity, well-being, or no disclosed problem, and when fear, performance anxiety, and episodic erectile difficulty were documented despite an IIEF-6 score above the exploratory threshold. Narrative silence denoted unavailable interview material, not the absence of concern.

**Figure 2:**
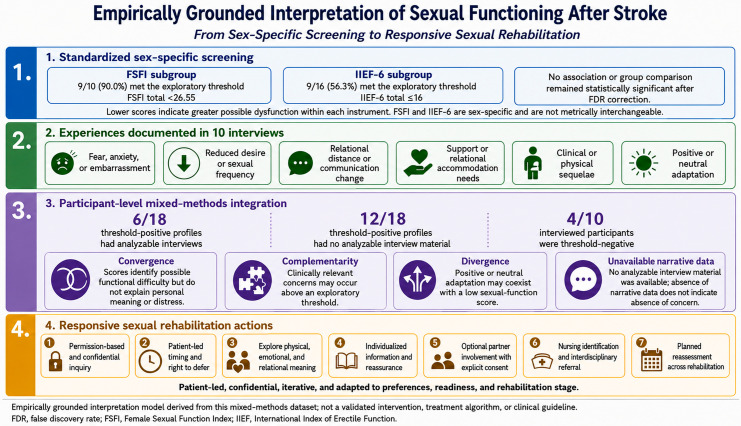
Empirically grounded interpretation of sexual functioning after stroke, from sex-specific screening to responsive sexual rehabilitation. The model integrates four findings from this dataset: (1) 9/10 FSFI profiles and 9/16 IIEF-6 profiles met the respective exploratory thresholds, with no FDR-significant association or group comparison; (2) 10 interviews documented fear or embarrassment, reduced desire or frequency, relational or communication change, support needs, clinical or physical sequelae, and positive or neutral adaptation; (3) participant-level integration showed 6/18 threshold-positive profiles with interviews, 12/18 with narrative silence, and 4/10 interviewed participants who were threshold-negative; and (4) these patterns support confidential, permission-based inquiry, patient-led timing, individualized information, optional partner involvement with explicit consent, interdisciplinary referral, and reassessment. The figure is an empirically grounded interpretation model, not a validated intervention, treatment algorithm, or clinical guideline. Abbreviations: FDR, false discovery rate; FSFI, Female Sexual Function Index; IIEF, International Index of Erectile Function.

These case-level patterns explain why the absence of FDR-significant associations cannot be equated with the absence of clinical relevance. Screening identified possible functional difficulty but did not determine subjective distress, relational meaning, or readiness to discuss sexuality. Conversely, narrative inquiry identified clinically relevant concerns that were not reducible to threshold status. Figure 2 synthesizes these empirically observed gaps and their implications for responsive sexual rehabilitation; it is an interpretation model, not a validated intervention, treatment algorithm, or clinical guideline.

## 4. Discussion

This convergent mixed-methods study showed that standardized sexual-function screening and documented narratives provided different but clinically complementary information after stroke. Exploratory thresholds identified possible dysfunction in 9 of 10 FSFI profiles and 9 of 16 IIEF-6 profiles, yet no association or group comparison remained statistically significant after FDR correction. The study’s contribution therefore lies not in detecting quantitative predictors but in demonstrating at the participant level how scores, subjective distress, relational meaning, support needs, positive adaptation, and missing narrative data may align or diverge. The findings remain preliminary and hypothesis-generating [38,39,40].

The participant-level joint display sharpened this interpretation. Only 6 of 18 threshold-positive profiles had analyzable interviews, whereas 12 represented narrative silence; four interviewed participants were threshold-negative. Within the available narratives, low scores could coexist with serenity, well-being, or no disclosed problem, while fear, performance anxiety, and episodic erectile difficulty occurred in a participant whose IIEF-6 score was above the exploratory threshold. These patterns do not invalidate screening. Rather, they show that a score can flag possible functional difficulty without establishing distress, post-stroke onset, personal meaning, or need for intervention, and that clinically relevant concerns may occur outside dichotomized threshold categories [41,42,43].

The sex-specific quantitative strand provided an essential boundary for interpretation. FSFI and IIEF-6 were kept separate because they assess different domains and are not metrically interchangeable; within-instrument ranks in Table 4 describe ordering only and cannot support cross-instrument severity comparisons. The largest uncorrected coefficients were reported for transparency, but all FDR-adjusted *p*-values were non-significant. Age, vascular burden, neurological injury, medication exposure, mood, disability, and relationship context may influence sexual functioning after stroke, but the present subgroups were not powered to test these determinants [44,45,46,47].

Participant-level integration distinguished four patterns. Convergence indicated alignment between screening and documented experience; complementarity indicated that narratives supplied meaning or support needs beyond the score; divergence indicated discordance between threshold status and disclosed experience; and narrative silence indicated unavailable interview material. Absence of FDR-significant associations therefore did not mean absence of concern, while a low score did not establish distress. Standardized screening and confidential narrative inquiry are complementary and non-substitutable sources of clinical information [48,49].

### 4.1. Clinical Meaning of Responsive Sexual Rehabilitation

In this study, responsive sexual rehabilitation is operationalized as a confidential, permission-based, patient-led, and iterative clinical process that combines sex-specific screening with narrative inquiry; explores physical, emotional, relational, and contextual meanings; provides individualized information and reassurance; and activates nursing or interdisciplinary support according to the patient’s preferences, readiness, and rehabilitation stage. The term does not denote a validated treatment protocol. It describes how rehabilitation teams can respond proportionately to what a patient chooses to disclose and can revisit the topic as needs change.

Operationalization begins with a private, nonjudgmental invitation that normalizes sexuality as a legitimate rehabilitation domain and explicitly permits the patient to decline or defer. When shame, embarrassment, or fear is present, clinicians should avoid assumptions, clarify preferred language and timing, and explore whether the concern relates to safety, bodily change, desire, performance, relationship dynamics, or access to information. Individualized reassurance or education can then be offered; partner involvement should remain optional and require explicit patient consent. Nurses may identify concerns across inpatient care, education, discharge, and follow-up, while physicians, psychologists, therapists, and other rehabilitation professionals can address medical, emotional, functional, or relational needs through defined referral pathways [50,51,52,53,54,55,56,57,58]. Planned reassessment is important because readiness may change. Figure 2 summarizes these actions in relation to the empirical integration patterns.

### 4.2. Strengths and Limitations

Several strengths should be acknowledged. The convergent design was suited to a topic in which numeric scores and personal meanings may not overlap, and FSFI and IIEF-6 were analyzed separately. Relational, family, depressive, quality-of-life, and clinical descriptors situated sexual health within a broader rehabilitation context. The participant-level joint display made the exact overlap between threshold status and available narratives transparent and distinguished convergence, complementarity, divergence, and narrative silence, consistent with mixed-methods reporting principles in nursing and health research [59].

The limitations are substantial. The total sample was small, and the sex-specific subgroups were very limited (FSFI *n* = 10; IIEF-6 *n* = 16), reducing statistical power and increasing the instability of correlations and group comparisons. No quantitative association or comparison remained statistically significant after FDR correction. The study was conducted in a single clinical rehabilitation setting and may not represent the broader post-stroke population. Participants were enrolled at heterogeneous time points after the event, and the clinical records included heterogeneous event descriptors. Stroke severity, cognitive status, fatigue, pain, premorbid sexual function, pre-stroke relationship quality, comorbid vascular conditions, medication effects, and detailed lesion characteristics could not be adequately controlled. Because pre-stroke sexual function was not assessed, the descriptive classification of possible dysfunction cannot establish whether difficulties were new after stroke or pre-existing. FSFI scores were reconstructed from domain-level values rather than original item-level responses, limiting independent verification of item-level scoring. The qualitative material consisted of systematically documented semi-structured interview responses rather than full verbatim transcripts; therefore, saturation, deep phenomenological interpretation, and formal inter-rater reliability could not be claimed. Partner or caregiver impressions were available only as contextual fields and not as an independent dyadic sample. Finally, the cross-sectional and exploratory design prevents causal inference.

Only 6 of the 18 threshold-positive profiles had analyzable interview material; the 12 cases of narrative silence constrained participant-level integration and prevented inference about the meaning or clinical relevance of their screening results.

Future studies should build on these descriptive mixed-methods observations. Larger multicenter and longitudinal cohorts are needed, but they should also collect richer narrative material, partner-level data, timing from stroke onset, premorbid sexual and relational information, comorbidity profiles, medication exposure, cognitive status, stroke severity, and validated item-level sex-specific scoring when feasible. Full verbatim interviews would help clarify how fear, attractiveness, body image, communication, desire, consent, caregiver roles, cultural norms, and positive adaptation evolve over time. Future intervention studies should test nurse-led or interdisciplinary approaches that combine permission-giving, individualized education, psychosocial support, and referral pathways while allowing patients to decide whether, when, and how sexuality is relevant to recovery. Such work would align with person-centered stroke care and contemporary rehabilitation models that extend beyond impairment reduction to participation, quality of life, and meaningful everyday roles [60,61].

## 5. Conclusions

Sexual functioning after stroke cannot be characterized adequately by either standardized screening or narrative discussion alone. In this small convergent mixed-methods study, FSFI and IIEF-6 threshold classifications identified possible sex-specific functional difficulty, while participant-level narratives differentiated distress, relational meaning, support needs, positive adaptation, and narrative silence. No quantitative association or group comparison remained statistically significant after FDR correction, and findings should remain separate by instrument and be interpreted as preliminary.

Responsive sexual rehabilitation is therefore best understood here as a clinical process, not a validated intervention, in which private, permission-based and patient-led inquiry combines sex-specific screening with individualized conversation, information, and, when desired, nursing or interdisciplinary support. Respect for preference, confidentiality, timing, the right to defer, optional partner involvement with explicit consent, and planned reassessment are central. These hypothesis-generating findings warrant larger longitudinal studies with premorbid sexual data, richer interviews, partner-level information, and control of clinical confounders before effectiveness or causal claims can be made.

## Figures and Tables

**Table 1:** Instruments and data sources used in the mixed-methods assessment of sexual functioning after stroke.

Domain Assessed	Instrument/Data Source	Main Variables or Domains Recorded	Study Role and Analytical Note
Sexual functioning (female)	Female Sexual Function Index (FSFI)	Desire, arousal, lubrication, orgasm, satisfaction, pain, and total score when applicable	Primary quantitative outcome for participants with female sexual function data; analyzed separately from male sexual function data
Sexual functioning (male)	IIEF-6 field recorded in the database	Total erectile function score	Primary quantitative outcome for participants with male erectile function data; not pooled with FSFI because the measures are not metrically interchangeable
Depressive symptoms	Hamilton Depression Rating Scale	Total score	Secondary quantitative outcome and correlate of sexual functioning
Health-related quality of life	12-item Short Form Health Survey (SF-12)	Physical, mental, and total scores	Secondary quantitative outcome and correlate of sexual functioning
Relationship functioning	Dyadic Adjustment Scale	Consensus, satisfaction, shared activities, affective and sexual life, and total score	Secondary relational outcome
Family relationship functioning	Family Relationship Index	Cohesion, communication, conflict, and total score	Secondary family-context outcome
Clinical descriptors	Study database fields	Age, event and enrollment dates, neuroimaging or event description, etiology code, medication exposure, education, and relationship variables	Used for sample characterization and exploratory covariates
Qualitative experiences	Semi-structured interview guide and documented responses	Sexuality changes, intimacy, identity, attractiveness, communication, barriers, rehabilitation needs, and partner/caregiver contextual perceptions	Qualitative component; analyzed descriptively and thematically

Abbreviations: FSFI, Female Sexual Function Index; IIEF, International Index of Erectile Function; SF-12, 12-item Short Form Health Survey. FSFI and IIEF-6 were analyzed separately because they are sex-specific measures and are not metrically interchangeable.

**Table 2:** Descriptive characteristics of the quantitative sample.

Variable	N	Median [IQR]
**Overall sample characteristics**		
Age (years)	26	48.50 [40.50–57.50]
Relationship duration (years)	26	9.00 [3.25–15.75]
Time from event to enrollment (months)	26	3.99 [2.40–10.76]
Year of schooling	26	13.00 [9.25–13.00]
DAS consensus on important issues	26	52.00 [45.50–57.75]
DAS satisfaction with relationship status	26	26.50 [19.50–28.75]
DAS shared activities	26	12.00 [8.25–14.75]
DAS satisfaction with affective and sexual life	26	11.50 [8.50–14.00]
DAS total	26	104.50 [83.50–114.75]
FRI family cohesion	26	4.00 [3.00–4.00]
FRI communication	26	3.00 [2.25–4.00]
FRI conflict	26	3.00 [2.00–4.00]
FRI total	26	10.00 [7.25–11.00]
HAM-D	26	12.50 [8.00–15.75]
SF-12 physical	26	12.50 [11.00–15.00]
SF-12 mental	26	19.00 [17.00–20.00]
SF-12 total	26	31.00 [28.00–34.00]
**Sex-specific sexual function measures**		
FSFI desire	10	3.60 [2.10–3.60]
FSFI arousal	10	0.90 [0.00–2.33]
FSFI lubrication	10	1.65 [0.00–3.60]
FSFI orgasm	10	1.80 [0.00–4.20]
FSFI satisfaction	10	0.60 [0.00–2.40]
FSFI pain	10	1.80 [0.00–5.90]
FSFI total	10	11.40 [3.15–21.75]
IIEF-6	16	10.00 [1.75–24.25]

Note: Values are presented as median [interquartile range]. FSFI domain and total scores were recalculated using standard FSFI domain weighting factors from domain-level data. The previous unweighted raw-sum total was not used for analysis, and item-level FSFI scoring could not be independently audited from the available database. **Abbreviations:** DAS, Dyadic Adjustment Scale; FRI, Family Relationship Index; FSFI, Female Sexual Function Index; HAM-D, Hamilton Depression Rating Scale; IIEF, International Index of Erectile Function; IQR, interquartile range; SF-12, 12-item Short Form Health Survey.

**Table 3:** Main qualitative themes identified in the analyzable narrative material and descriptive verification strategy.

Theme	Meaning Unit and Theme Description	Supporting Clinical Material
Emotional discomfort	Fear, anxiety, embarrassment, or performance-related concern during sexual relations	“Fear and performance anxiety”; “Embarrassment”
Reduced sexual desire	Reduced sexual interest after stroke or during the post-stroke period	“Low sexual interest”; “Reduced sexual desire”
Reduced sexual frequency	Decrease in the frequency of sexual intercourse	“Lower frequency of sexual intercourse”
Relational distance/change	Changes in closeness, communication, or couple dynamics	“Greater distance”; “No longer takes the time to explain things…”
Need for relational support	Need for greater collaboration, understanding, or accommodation	“Need to be more collaborative”
Clinical/physical sequelae	Motor, autonomic, or functional consequences acting as contextual factors	“Motor difficulties”; “Constipation”; “Difficulty achieving and/or maintaining an erection”
Positive or neutral adaptation	Serenity, tranquility, well-being, or absence of reported sexual problems	“Serenity”; “Calmness”; “No problems reported”

Note: Themes were derived by grouping clinically meaningful units from systematically documented semi-structured interview responses. Candidate themes were checked against the source entries and discussed by the analytic team until descriptive consensus was reached. Because the material consisted of structured case report forms rather than verbatim interview transcripts, no formal saturation claim or inter-rater reliability coefficient was calculated; credibility was supported through transparent theme definitions, illustrative examples, and explicit limitation of the interpretive scope.

**Table 4:** Participant-level mixed-methods joint display linking sex-specific sexual-function scores with available semi-structured interview material.

ID	Scale	Total Score	Threshold Status	Within-Scale Rank	Interview	Documented Theme(s)	Integration Pattern	Case-Level Interpretation
P23	FSFI	1.2	Threshold-positive (<26.55)	1/10 (tie)	Yes	Relational distance/change; reduced sexual frequency; desire concern in pre-event/partner contextual fields; indifference	Convergence/complementarity	Very low score aligned with relational distance and reduced frequency; timing of the desire concern was uncertain and could not be attributed to stroke.
P24	FSFI	1.2	Threshold-positive (<26.55)	1/10 (tie)	Yes	Well-being/tranquility; no specific concern disclosed	Divergence	Very low score coexisted with positive or neutral adaptation; score alone could not establish distress or post-stroke onset.
P11	FSFI	3.0	Threshold-positive (<26.55)	3/10	No	No analyzable interview response was available.	Narrative silence	Threshold-positive screening; unavailable narrative material prevented inference about meaning, distress, or support need.
P04	FSFI	3.6	Threshold-positive (<26.55)	4/10 (tie)	No	No analyzable interview response was available.	Narrative silence	Threshold-positive screening; unavailable narrative material prevented inference about meaning, distress, or support need.
P22	FSFI	3.6	Threshold-positive (<26.55)	4/10 (tie)	Yes	Reduced sexual frequency; embarrassment; reported relationship improvement/relational accommodation; tranquility/no specific problem	Divergence/complementarity	Very low score coexisted with tranquility and no disclosed problem; frequency and relational entries added context without establishing cause.
P10	FSFI	19.2	Threshold-positive (<26.55)	6/10	No	No analyzable interview response was available.	Narrative silence	Threshold-positive screening; unavailable narrative material prevented inference about meaning, distress, or support need.
P19	FSFI	21.6	Threshold-positive (<26.55)	7/10	Yes	Tranquility; no relationship/sexual change or specific concern disclosed	Divergence	Threshold-positive score coexisted with neutral adaptation and no disclosed concern; screening did not determine subjective distress.
P07	FSFI	21.8	Threshold-positive (<26.55)	8/10	No	No analyzable interview response was available.	Narrative silence	Threshold-positive screening; unavailable narrative material prevented inference about meaning, distress, or support need.
P17	FSFI	24.0	Threshold-positive (<26.55)	9/10	Yes	Reduced desire; reduced sexual frequency; serenity	Convergence/complementarity	Score aligned with documented desire and frequency concerns, while serenity showed that distress could not be inferred from the score alone.
P25	FSFI	28.0	Threshold-negative (≥26.55)	10/10	Yes	Serenity; no specific concern disclosed; reported frequency change	Convergence/complementarity	Above-threshold score aligned with no disclosed problem; frequency history added contextual information.
P03	IIEF-6	1	Threshold-positive (≤16)	1/16 (tie)	No	No analyzable interview response was available.	Narrative silence	Threshold-positive screening; unavailable narrative material prevented inference about meaning, distress, or support need.
P12	IIEF-6	1	Threshold-positive (≤16)	1/16 (tie)	No	No analyzable interview response was available.	Narrative silence	Threshold-positive screening; unavailable narrative material prevented inference about meaning, distress, or support need.
P13	IIEF-6	1	Threshold-positive (≤16)	1/16 (tie)	No	No analyzable interview response was available.	Narrative silence	Threshold-positive screening; unavailable narrative material prevented inference about meaning, distress, or support need.
P14	IIEF-6	1	Threshold-positive (≤16)	1/16 (tie)	No	No analyzable interview response was available.	Narrative silence	Threshold-positive screening; unavailable narrative material prevented inference about meaning, distress, or support need.
P08	IIEF-6	2	Threshold-positive (≤16)	5/16 (tie)	No	No analyzable interview response was available.	Narrative silence	Threshold-positive screening; unavailable narrative material prevented inference about meaning, distress, or support need.
P15	IIEF-6	2	Threshold-positive (≤16)	5/16 (tie)	No	No analyzable interview response was available.	Narrative silence	Threshold-positive screening; unavailable narrative material prevented inference about meaning, distress, or support need.
P16	IIEF-6	5	Threshold-positive (≤16)	7/16	No	No analyzable interview response was available.	Narrative silence	Threshold-positive screening; unavailable narrative material prevented inference about meaning, distress, or support need.
P05	IIEF-6	7	Threshold-positive (≤16)	8/16	No	No analyzable interview response was available.	Narrative silence	Threshold-positive screening; unavailable narrative material prevented inference about meaning, distress, or support need.
P26	IIEF-6	13	Threshold-positive (≤16)	9/16	Yes	Relational communication change; reported frequency change; well-being/no specific concern	Divergence/complementarity	Threshold-positive score coexisted with well-being and no disclosed sexual concern; narrative material added relational and frequency context.
P18	IIEF-6	22	Threshold-negative (>16)	11/16	Yes	Serenity; no specific concern; reported frequency change	Convergence/complementarity	Above-threshold score aligned with no disclosed concern; narrative material provided adaptive context.
P21	IIEF-6	24	Threshold-negative (>16)	13/16	Yes	Fear/performance anxiety; erectile difficulty; reduced frequency; cardiopathy/low blood pressure as clinical context	Divergence/complementarity	Score above the conservative threshold did not exclude clinically relevant concern; interview material captured fear and episodic erectile difficulty.
P20	IIEF-6	30	Threshold-negative (>16)	16/16	Yes	Reduced sexual frequency; embarrassment; well-being/no post-event problem disclosed	Divergence/complementarity	High IIEF-6 score coexisted with frequency decline and embarrassment, illustrating information missed by dichotomization.

Note: One pseudonymized row is shown for each participant who met an exploratory threshold and/or had analyzable interview material (22 unique cases). FSFI totals were reconstructed from domain-level raw sums using standard weighting factors; item-level responses were unavailable. Thresholds were FSFI total < 26.55 and IIEF-6 total ≤ 16. Lower scores indicate greater possible dysfunction within each instrument only. Ascending ranks were calculated within the complete FSFI (n = 10) or IIEF-6 (n = 16) subgroup; ties share rank, and no cross-instrument severity comparison is valid. Threshold-positive denotes an exploratory screening classification, not a diagnosis. Narrative silence denotes unavailable analyzable interview material, not absence of concern. Partner/caregiver entries were used only as contextual fields, not as an independent dyadic sample. Integration patterns are descriptive and were not subjected to inferential mixed-methods testing. Abbreviations: FSFI, Female Sexual Function Index; IIEF, International Index of Erectile Function.

## Data Availability

The data presented in this study are not publicly available because they contain sensitive clinical and sexual-health information. De-identified data may be made available from the corresponding author upon reasonable request and subject to applicable ethical and privacy restrictions.

## Supplementary Materials

The following supporting information can be downloaded at: https://www.mdpi.com/article/10.3390/nursrep16070243/s1, Figure S1: Simplified qualitative theme map of post-stroke sexual and relational experiences; Table S1: Additional categorical characteristics of the quantitative sample; Table S2: Group-wise descriptive summaries for exploratory comparisons of sex-specific total sexual-function scores; Table S3: Exploratory nonparametric group comparisons of sex-specific total sexual-function scores.

## Public Involvement Statement

No public involvement in any aspect of this research.

## Guidelines and Standards Statement

This manuscript was drafted with attention to the Strengthening the Reporting of Observational Studies in Epidemiology (STROBE) statement for the quantitative component, the Consolidated Criteria for Reporting Qualitative Research (COREQ) for the descriptive interview component, and the Good Reporting of A Mixed Methods Study (GRAMMS) principles for mixed-methods reporting [35,36,37].

## Use of Artificial Intelligence

The authors acknowledge the use of the latest available version of ChatGPT (OpenAI, GPT-5.5 Thinking model, accessed through the ChatGPT web interface) solely for assistance with figure conceptualization, visual layout, and graphical formatting. No artificial-intelligence tool was used for data generation, data analysis, statistical processing, qualitative interpretation, or scientific decision-making. The authors reviewed and verified all AI-assisted visual materials and remain fully responsible for the content of the manuscript.
