# Supplementary material for: Sexual Functioning and Patient-Reported Concerns After Stroke: An Integrated Mixed-Methods Study in Clinical Rehabilitation
Source: Nurs Rep. 2026 Jul 14;16(7):243. doi: 10.3390/nursrep16070243 (PMC13414991; doi:10.3390/nursrep16070243)
Supplement: Supplementary file 1 [file nursrep-16-00243-s001.zip › nursrep-4346104-supplementary.pdf]

Supplementary Materials

Sexual Functioning and Patient-Reported Concerns After Stroke: An Integrated Mixed-Methods Study in Clinical Rehabilitation

Table S1. Additional categorical characteristics of the quantitative sample

Characteristic	n	%
Etiological code 1	11	42.31%
Etiological code 2	15	57.69%
Pharmacological treatment: Yes	15	57.69%
Pharmacological treatment: No	7	26.92%
Pharmacological treatment: Missing	4	15.38%
Lesion side: Right-sided	13	50.00%
Lesion side: Left-sided	5	19.23%
Lesion side: Unclear/not specified	8	30.77%
Broad event type: Likely ischemic/other	17	65.38%
Broad event type: Hemorrhagic/hematoma	5	19.23%
Broad event type: Post-traumatic/stroke-related	1	3.85%
Broad event type: Unclear clinical descriptor	3	11.54%
FSFI data available	10	38.46%
IIEF-6 data available	16	61.54%

Note: Lesion side and broad event type were derived from narrative clinical descriptors and are reported for descriptive purposes only. Percentages are calculated using the full quantitative sample (N = 26).

Table S2. Group-wise descriptive summaries for exploratory comparisons of sex-specific total sexual-function scores

Outcome	Grouping variable	Category	n	Median [IQR]
FSFI total	Overall FSFI subgroup	All	10	11.40 [3.15–21.75]
IIEF-6 total	Overall IIEF-6 subgroup	All	16	10.00 [1.75–24.25]
FSFI total	Etiological code	Code 1	5	3.60 [1.20–24.00]
FSFI total	Etiological code	Code 2	5	19.20 [3.60–21.60]
FSFI total	Pharmacological treatment	No	1	24.00 [24.00–24.00]
FSFI total	Pharmacological treatment	Yes	9	3.60 [3.00–21.60]
FSFI total	Lesion side from narrative field	Left-sided	3	1.20 [1.20–2.40]
FSFI total	Lesion side from narrative field	Right-sided	2	24.90 [23.35–26.45]
FSFI total	Lesion side from narrative field	Unclear/not specified	5	19.20 [3.60–21.60]
FSFI total	Broad event type from narrative field	Hemorrhagic/hematoma	2	12.70 [8.15–17.25]
FSFI total	Broad event type from narrative field	Likely ischemic/other	6	11.40 [1.80–22.80]
FSFI total	Broad event type from narrative field	Unclear clinical descriptor	2	12.30 [7.65–16.95]
FSFI total	Civil status	Code 0	2	2.10 [1.65–2.55]
FSFI total	Civil status	Code 1	1	24.00 [24.00–24.00]
FSFI total	Civil status	Code 2	1	21.60 [21.60–21.60]

Outcome	Grouping variable	Category	n	Median [IQR]
FSFI total	Civil status	Code 3	6	11.40 [3.60–21.15]
FSFI total	Parental status	Code 0	3	3.00 [2.10–13.50]
FSFI total	Parental status	Code 1	2	24.80 [23.20–26.40]
FSFI total	Parental status	Code 2	5	3.60 [3.60–19.20]
IIEF-6 total	Etiological code	Code 1	6	12.00 [1.25–23.50]
IIEF-6 total	Etiological code	Code 2	10	10.00 [2.75–23.50]
IIEF-6 total	Pharmacological treatment	No	6	16.00 [3.25–25.00]
IIEF-6 total	Pharmacological treatment	Yes	6	20.50 [14.50–23.50]
IIEF-6 total	Pharmacological treatment	Missing	4	1.50 [1.00–2.75]
IIEF-6 total	Lesion side from narrative field	Left-sided	2	3.50 [2.75–4.25]
IIEF-6 total	Lesion side from narrative field	Right-sided	11	19.00 [4.00–25.00]
IIEF-6 total	Lesion side from narrative field	Unclear/not specified	3	2.00 [1.50–12.00]
IIEF-6 total	Broad event type from narrative field	Hemorrhagic/hematoma	3	7.00 [4.50–10.00]
IIEF-6 total	Broad event type from narrative field	Likely ischemic/other	11	19.00 [1.50–25.00]
IIEF-6 total	Broad event type from narrative field	Post-traumatic/stroke-related	1	1.00 [1.00–1.00]
IIEF-6 total	Broad event type from narrative field	Unclear clinical descriptor	1	22.00 [22.00–22.00]
IIEF-6 total	Civil status	Code 0	4	16.00 [5.75–26.25]
IIEF-6 total	Civil status	Code 1	4	11.50 [1.00–22.75]
IIEF-6 total	Civil status	Code 2	1	13.00 [13.00–13.00]
IIEF-6 total	Civil status	Code 3	5	19.00 [5.00–24.00]
IIEF-6 total	Civil status	Code 4	2	1.50 [1.25–1.75]
IIEF-6 total	Parental status	Code 0	6	23.50 [10.75–25.00]
IIEF-6 total	Parental status	Code 1	3	5.00 [3.00–9.00]
IIEF-6 total	Parental status	Code 2	7	2.00 [1.00–21.50]

Note. FSFI totals were reconstructed from domain-level values using standard domain weighting factors. IIEF-6 totals were taken from the database field. These summaries are descriptive only and should not be interpreted as subgroup effects. Categories labelled as codes retain the source-database coding.

Table S3. Exploratory nonparametric group comparisons of sex-specific total sexual-function scores

Outcome	Grouping variable	Test	Effect size	p-value	pFDR	Interpretation
FSFI total	Etiological code	Wilcoxon rank-sum	$r = -0.08$	0.916	0.916	Not statistically significant after FDR correction
FSFI total	Pharmacological treatment	Wilcoxon rank-sum	$r = 0.78$	0.293	0.440	Not statistically significant after FDR correction
FSFI total	Lesion side	Kruskal–Wallis	$\varepsilon^2 = 0.54$	0.056	0.334	Not statistically significant after FDR correction
FSFI total	Broad event type	Kruskal–Wallis	$\varepsilon^2 = 0.00$	0.912	0.916	Not statistically significant after FDR correction
FSFI total	Civil status	Kruskal–Wallis	$\varepsilon^2 = 0.16$	0.264	0.440	Not statistically significant after FDR correction
FSFI total	Parental status	Kruskal–Wallis	$\varepsilon^2 = 0.07$	0.284	0.440	Not statistically significant after FDR correction
IIEF-6 total	Etiological code	Wilcoxon rank-sum	$r = -0.12$	0.742	0.872	Not statistically significant after FDR correction
IIEF-6 total	Pharmacological treatment	Wilcoxon rank-sum	$r = 0.08$	0.872	0.872	Not statistically significant after FDR correction
IIEF-6 total	Lesion side	Kruskal–Wallis	$\varepsilon^2 = 0.00$	0.452	0.807	Not statistically significant after FDR correction
IIEF-6 total	Broad event type	Kruskal–Wallis	$\varepsilon^2 = 0.00$	0.529	0.807	Not statistically significant after FDR correction
IIEF-6 total	Civil status	Kruskal–Wallis	$\varepsilon^2 = 0.00$	0.538	0.807	Not statistically significant after FDR correction
IIEF-6 total	Parental status	Kruskal–Wallis	$\varepsilon^2 = 0.13$	0.153	0.807	Not statistically significant after FDR correction

Note. Wilcoxon rank-sum tests were used for two-group comparisons, with effect size reported as rank-biserial correlation (r). Kruskal–Wallis tests were used for comparisons involving more than two groups, with effect size reported as epsilon-squared (ε^2). Missing pharmacological-treatment values were shown descriptively in Table S2 but excluded from the inferential No-versus-Yes comparison. The Benjamini–Hochberg procedure was applied separately to the six prespecified group comparisons within each sex-specific outcome. pFDR denotes the resulting FDR-adjusted p-value. Values are rounded to three decimal places; no comparison remained statistically significant after FDR correction. Abbreviations: FSFI, Female Sexual Function Index; IIEF, International Index of Erectile Function; FDR, false discovery rate.

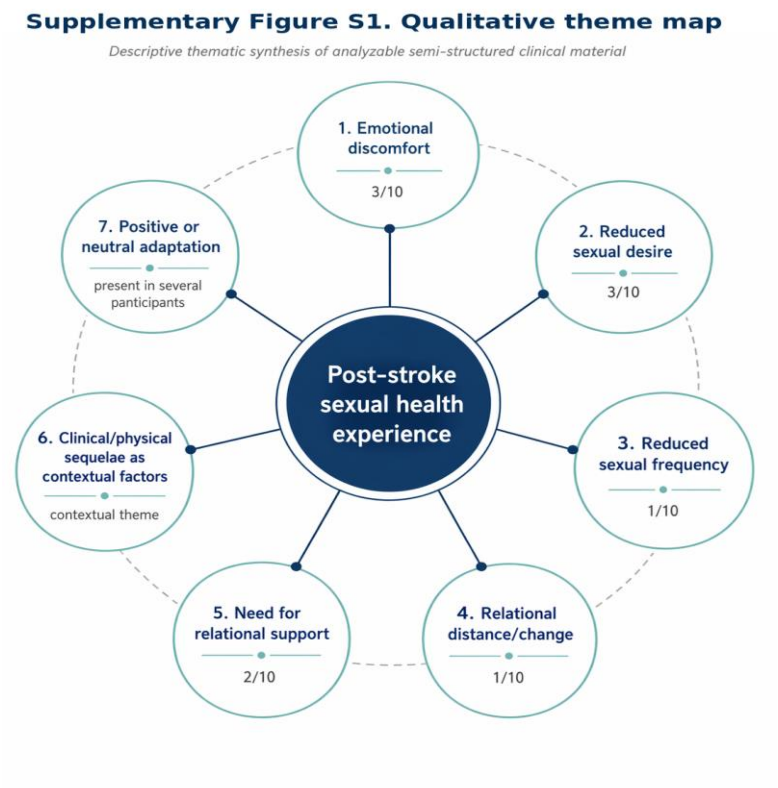

Supplementary Figure S1. Simplified qualitative theme map of post-stroke sexual and relational experiences.

The figure presents the main themes identified in the analyzable semi-structured clinical material. Themes are shown as interrelated dimensions of post-stroke sexual health experience rather than as mutually exclusive categories. Participant counts indicate the number of individuals in the qualitative subset in whom each theme was identified when a frequency could be assigned. Clinical and physical sequelae were treated as contextual factors unless explicitly linked to sexual functioning in the participant response. Positive or neutral adaptation was retained to avoid a solely deficit-oriented interpretation. The qualitative material consisted of brief semi-structured clinical entries rather than full verbatim interview transcripts.
